# CD8^+^, HLA-unrestricted, cytotoxic T-lymphocyte line against malignant melanoma

**DOI:** 10.1186/1479-5876-3-41

**Published:** 2005-11-10

**Authors:** Rajasekharan Somasundaram, Laura Caputo, DuPont Guerry, Dorothee Herlyn

**Affiliations:** 1 The Wistar Institute, 3601 Spruce Street, Philadelphia, PA 19104, USA; 2 Hematology-Oncology Division, Department of Medicine, at the Hospital of the University of Pennsylvania, 34^*th *^and Spruce Streets, Philadelphia, PA 19104, USA

## Abstract

A CD8^+ ^cytotoxic T lymphocyte (CTL) line was derived from the peripheral blood mononuclear cells of a patient with primary melanoma. The CD8^+ ^CTL line specifically lysed the autologous primary melanoma cells and not the natural killer cell-sensitive K562 cells or lymphokine activated killer cell-sensitive DAUDI cells. When a large panel of human leukocyte antigen (HLA)-matched and -unmatched allogeneic melanoma, glioma, breast and colorectal carcinoma cells was tested as targets in cytolysis assays, 4 HLA-matched and two HLA-unmatched allogeneic metastatic melanoma lines were lysed by the CD8^+ ^CTL. Lysis of autologous and allogeneic melanoma cells was dependent on the effector-to-target cell ratio. Lysis of autologous melanoma cells was not blocked by anti-HLA class I or class II antibodies, confirming that the cytolytic activity of the CD8^+ ^CTL was HLA-unrestricted. CTL lysis of autologous melanoma cells was CD3 (T cell receptor) dependent and FAS-FAS-L, and CD1 independent. Identification of the melanoma-associated antigen recognized by the HLA-unrestricted CTL may provide a vaccine for a broad population of melanoma patients.

## Background

Cytotoxic T lymphocytes (CTL) have been established from various lymphoid sources of patients with metastatic melanoma[1]. The majority of CTL are of CD8 phenotype and generally lyse tumor cells in a human leukocyte antigen (HLA)-restricted manner. A number of melanoma-associated antigens and peptides defined by CD8^+ ^CTL have been identified and the majority of these CTL are HLA-A2 restricted [1,2]. CD8^+ ^CTL that recognized and lysed melanoma targets of various HLA types also have been described [3-6]. However, it is unclear whether the CTL are truly HLA-unrestricted, as the allogeneic tumor target cells used were not fully HLA subtyped [3-6] and, therefore, partial HLA matching of these targets with the CTL cannot be excluded with certainty. Furthermore, the demonstration of the absence of HLA restriction was based on the absence of CTL lysis blocking in the presence of high concentrations of anti-HLA antibodies. However, the high effector-to-target (E:T) cell ratios used in those studies, accompanied by high tumor cell lysis in control cultures [5,6] may be responsible for the absence of CTL blocking by anti-HLA antibodies.

We have established a CD8^+ ^CTL line from the peripheral blood lymphocytes of a patient with primary melanoma. When the cytotoxic activity of the CTL line was tested against a large panel of allogeneic cell lines of melanoma, glioma, breast or colorectal carcinoma, autologous or allogeneic Epstein-Barr virus (EBV)-transformed B cells, or autologous fibroblasts, the autologous and a few allogeneic, HLA non-matched melanoma cells were lysed and induced interferon (IFN)-γ and granulocyte monocyte-colony stimulating factor (GM-CSF) secretion by the CTL. Furthermore, the lysis of the autologous or allogeneic tumor cells was not blocked by monoclonal antibodies (MAbs) to HLA-class I or class II, confirming that the CTL lyse targets in a HLA-unrestricted manner.

## Methods

### Patient 793

Patient 793 (male Caucasian, 39 years old) had excision of a "low risk" primary melanoma [superficial spreading type with early vertical growth phase present; the tumor thickness was 0.55 mm and the vertical growth phase had a brisk lymphoid infiltrate, with no evidence of metastases]. The primary lesion was excised ~20 years ago and there has been no recurrence since. The patient did not receive adjuvant chemotherapy after removal of the primary lesion.

### Cell lines

Melanoma cell line WM793 was established from the vertical growth phase of a primary lesion of patient 793 [7]. Cell line 1205LU, the metastatic variant of WM793, was established after repeated passages of WM793 cells both *in vitro *and *in vivo *in nude mice [8]. Melanoma cell lines WM75, WM98, WM164, and WM1158 were derived from metastatic lesions of melanoma patients [7]. All cell lines were maintained in McCoy's Dulbecco 153-Leibovitz 15 (MCDB153-L15) medium (Sigma, St. Louis, MO) containing 2% fetal bovine serum (FBS). Metastatic melanoma cell line DM196 was obtained from T. L. Darrow (Duke University Medical Center, Durham, NC) and was maintained in Dulbecco's modified Eagle's medium (DMEM; GIBCO-Invitrogen, Carlsbad, CA) supplemented with 5% FBS. Metastatic melanoma cell line ME9874 was obtained from A. Anichini (Istituto Nazionale Tumori, Milan, Italy) and maintained in RPMI 1640 Glutamax medium (GIBCO-Invitrogen) supplemented with 10% FBS. Metastatic melanoma cell line A375 was obtained from American Type Culture Collection (ATCC; Rockville, MD) and maintained in RPMI 1640 medium supplemented with 10% FBS. Rectal carcinoma cell lines WC007 and WC008 were maintained in MCDB20l-L15 (Sigma) medium supplemented with 2% FBS[9]. The glioma cell lines U373MG and U87MG (obtained from Dr. Darell Bigner, Duke University Medical Center) and the breast cancer cell line MDA MB231 (obtained from Dr. Daniela Santoli, The Wistar Institute) were maintained in DMEM medium supplemented with 10% FBS. HLA class I and II expression by tumor cells was determined in complement-dependent lysis assay using HLA typing trays (One Lambda, Inc., Canoga Park, CA). 793 fibroblast cell line [10] was maintained in DMEM containing 10% FBS.

EBV-transformed B cell line was established from freshly isolated peripheral blood mononuclear cells (PBMC) of patient 793 as described before[10]. EBV-B cell lines 888, 1363, 1088, 1102, and 4226 were established from melanoma patients' PBMC. Natural Killer (NK) cell target K562 (human erythroleukemia cell line) and lymphokine-activated killer (LAK) cell target Daudi (human lymphoblastoid cell line) were obtained from ATCC. All lymphoid cell lines were maintained in RPMI 1640 medium supplemented with 10% FBS.

### Antibodies

The following MAbs were used: HLA-class I-specific MAb W6/32 and HLA-class II-specific MAbs B33.1 and D1.B6 (obtained from B. Perussia, Thomas Jefferson University); MAb H24B5 to influenza virus hemagglutinin (obtained from W. Gerhard, The Wistar Institute); MAb ME36.1 to GD2 and GD3; MAb ME50.8 to GD3; MAb 31.3 to chondroitin sulfate proteoglycan; MAb MENu4B to v(e.c.)itronectin receptor; MAb 20.4 to nerve growth factor receptor; MAb 425 to epidermal growth factor receptor; MAb 451-1-C7-1 to fibroblast marker; MAbs 439-3-27-2-3 and 456-1-A11-2 to carbohydrates; MAbs ME7529 and ME77.1 to 120 kd protein on melanoma cells and MAb 507-2-2-4 to uncharacterized melanoma-associated antigen [11]. MAb HIT3a to CD3; MAb RPA-T4 to CD4; MAb RPA-T8 to CD8; MAb M-A251 to CD25; MAb 5C3 to CD40; MAb TRAP1 to CD40L; MAb L307.4 to B7-1 (CD80); MAb 2331 to B7-2 (CD86); MAb HI149 to CD1a; MAb Nok-1 to CD95L (Fas ligand), and MAb DX2 to CD95 (Fas receptor) (all antibodies were obtained from BD Pharmingen, San Diego, CA); MAb BMAO 31 to human T-cell receptor (TCR) α/β common region (T cell Sciences, Cambridge, MA); MAb OKT3 to CD3; MAbs 4F-2 and 5AG to IL-4 (DNAX, Palo Alto, CA); MAbs B133.1.1 and B133.5.1 to IFN-γ; MAbs B154.9.1 and B154.9.2 to tumor necrosis factor (TNF-α) (obtained from G. Trinchieri, The Wistar Institute); and MAb C9.1 and C16.3 to granulocyte macrophage-colony stimulation factor (GM-CSF) [12].

### Generation of anti-melanoma CTL line

CTL were obtained from co-cultures of PBMC (10^5 ^cells/well of 96-well round-bottom microtiter plates) of melanoma patient 793 with irradiated (40,000 rads, Cs source) autologous melanoma cells WM793 (10^5 ^cells/well) in RPMI 1640 medium containing 10% human AB serum (Sigma), 10 mm HEPES (Sigma), L-arginine (116 mg/l; GIBCO-Invitrogen), L-asparagine (36 mg/l; GIBCO-Invitrogen), L-glutamine (216 mg/l; Invitrogen), and 2-mercaptoethanol (ME) (5 × 10^-5 ^M; Sigma). Cultures were periodically stimulated with irradiated autologous tumor cells and irradiated autologous PBMC (days 8 and 14) or autologous EBV-B cells (from day 21 on) in RPMI 1640 medium containing partially purified IL-2 (day 3 on; 20 U/ml; Advanced Biotechnologies., Columbia, MD).

### Cytotoxicity assay

This assay was performed as described previously [9,10,13]. Briefly, labeled targets [2 μCi of ^51^Cr (sodium chromate, specific activity 400–1200 Ci/g; NEN DuPont, Boston, MA) per 1 × 10^4 ^cells] were mixed in 96-well U-bottom microtiter plates with effector cells at various E:T ratios and incubated at 37°C for 4 hr. Supernatants were harvested and tested for ^51^Cr release (experimental release). For maximal release, target cells were treated with 0.3% Triton X-100 (Sigma). Spontaneous release of radioactivity by target cells was determined in the absence of effector cells. Spontaneous release of the various targets used was 10–15%. The percentage of cytotoxicity was determined by the following formula:



The targets (WC008, U373MG and U87MG) that exhibited high spontaneous ^51^Cr release were stained with neutral red dye and used in a modified cytotoxicity assay[10].

### Blocking of cytotoxic activity

Cytotoxicity blocking assays were performed as described before [10,13]. Briefly, tumor targets were incubated with 10 μg/ml of anti-HLA-class I MAb W6/32 (IgG2a) or anti-HLA-class II MAb B33.1 and D1.B6 (IgG2a), MAb DX2 (IgG1) to Fas or HI149 (IgG1) to CD1, and CTL were incubated with 10 μg/ml of anti-CD3 MAb OKT3 (IgG2a). Isotype-matched control MAb H24B5 (IgG2a) or normal mouse IgG (predominantly IgG1) were used at 10 μg/ml. All incubations were performed for 1 hr at room temperature. Following blocking of tumor cells or CTL, cytotoxicity assays were performed as described above.

### Cytokine determinations

Cultured CTL were washed twice with serum-containing RPMI 1640 medium, incubated in serum-containing medium (without stimulants or IL-2) for 10–12 hr at 37°C in a 5% CO_2 _incubator and washed once. CTL (10^4 ^cells/well) were stimulated with irradiated autologous or allogeneic tumor or normal cells (10^4^–10^5 ^cells/well) in 96-well microtiter plates. Supernatants obtained from cultured CTL after 24 hr or 72 hr were tested for the presence of GM-CSF, TNF-α, IL-4, and IFN-γ.

IL-4, IFN-γ, TNF-α, and GM-CSF were measured by radioimmunoassay (RIA) as described before. [9] T cell supernatants at various dilutions were placed in antibody-coated wells (MAb 4F-2 to IL-4; MAb B133.1.1 to IFN-γ; MAb B154.9.2 to TNF-α; MAb C9.1 to GM-CSF), and binding was determined using ^125^I-labeled MAb specific for different determinants on the cytokines (MAb 5AG to IL-4; MAb B133.5.1 to IFN-γ; MAb B154.9.1 to TNF-α; MAb C16.3 to GM-CSF). Concentrations of IL-4, IFN-γ, TNF-α, and GM-CSF were determined using respective recombinant cytokine standard preparations (R&D Systems Inc., Minneapolis, MN).

IL-2 expression by the T cells was tested by reverse transcriptase-polymerase chain reaction (RT-PCR) as described[10].

### Phenotyping of melanoma and T cells

Cultured cells were incubated with saturating concentrations (5 μg/ml) of either unlabeled or fluoresceinated or phycoerythrin(PE)-labeled MAbs detecting human lymphocyte markers (CD3, CD4, CD8, CD25, CD40, CD40L, CD80, CD86, CD95, and CD95L) or melanoma-associated antigens (ME36.1, ME50.8, 31.3, MENu4B, 20.4, 425, 451-1-C7-1, 439-3-27-2-3, 456-1-A11-2, ME7529, 77.1 and 507-2-2-4) in RPMI 1640 medium supplemented with 5% human AB serum for 45 min at 4°C. Cells were washed 3× and stained with fluorescein isothiocyanate conjugated (FITC) second antibody [goat anti-mouse F(ab)_2_, 1:200 dilution, ICN-Cappel, Irvine, CA]. Binding of the PE or FITC-conjugated antibodies was analyzed in the cytofluorograph (Ortho Diagnostics, Rariton, NJ). All values given in "Results" are corrected for irrelevant, isotype-matched control antibody binding.

### TCR analysis by RT-PCR

TCR was analyzed as describted previously[10]. In brief, mRNA was extracted from 3 × 10^6 ^T cells using the mRNA DIRECT kit (Dynal Biotech, Lake Success, NY). RT-PCR and cDNA synthesis were performed using the SuperScript One-Step RT-PCR kit (Invitrogen, Carlsbad, CA), specific 5' primers encoding variable Vα (1–22) and Vβ (1–24) and a common C region (Cα and Cβ) 3' primer. PCR products were run on 2% agarose gels and analyzed.

### Statistical analyses

Differences between experimental and control values (triplicates or quadruplicates) were analyzed for significance by Student's two-sided paired t-test.

## Results

### Phenotypic characteristics of T cell line

A T cell line was generated in mixed lymphocyte/tumor culture (MLTC) by stimulating the PBMC of melanoma patient 793 with irradiated autologous WM793 melanoma cells. Cultures received IL-2 from day 3 on and were restimulated with irradiated autologous tumor cells and IL-2 every week. The T cell line was >98% CD8^+ ^(Table I). The CD8^+ ^T cell line expressed HLA class I and II, CD3, CD25, FAS, FASL, B7-1, and B7-2, and a low percentage of the cells also expressed CD40 and CD40L (Table I). The T cell line expressed TCR α/β chains. Further, RT-PCR analysis indicated that the T cell line is oligoclonal as it expresses Vα2, Vα4, Vα14, Vα16 and Vβ6 chains. Attempts to clone CD8^+ ^T cell line by limiting dilution were unsuccessful twice. WM793 melanoma cells expressed HLA class I and II and FAS, and a low percentage of the cells expressed FASL, B7-1, and B7-2 (Table I).

**Table 1:** Phenotypic markers of CD8^+ ^CTL793 and WM793 Melanoma Cells

	Percent cells positive	
	
Parameter investigated	CD8^+ ^CTL793	WM793 melanoma cells
HLA Class I	95	97
HLA Class II	95	74
CD1	NA^a^	18
CD3	90	NA
CD4	0	NA
CD8	98	NA
CD25	80	0
CD95 (FAS)	98	95
CD95L (FAS-L)	89	10
CD80 (B7-1)	88	7
CD86 (B7-2)	32	3
CD40	10	3
CD40L	18	6
TCRα/β	92	NA

^a ^NA = not applicable.

### Cytolytic activity of CD8^+ ^CTL line against various HLA-matched and non-matched tumor and non-tumor cells

**Figure 1:**
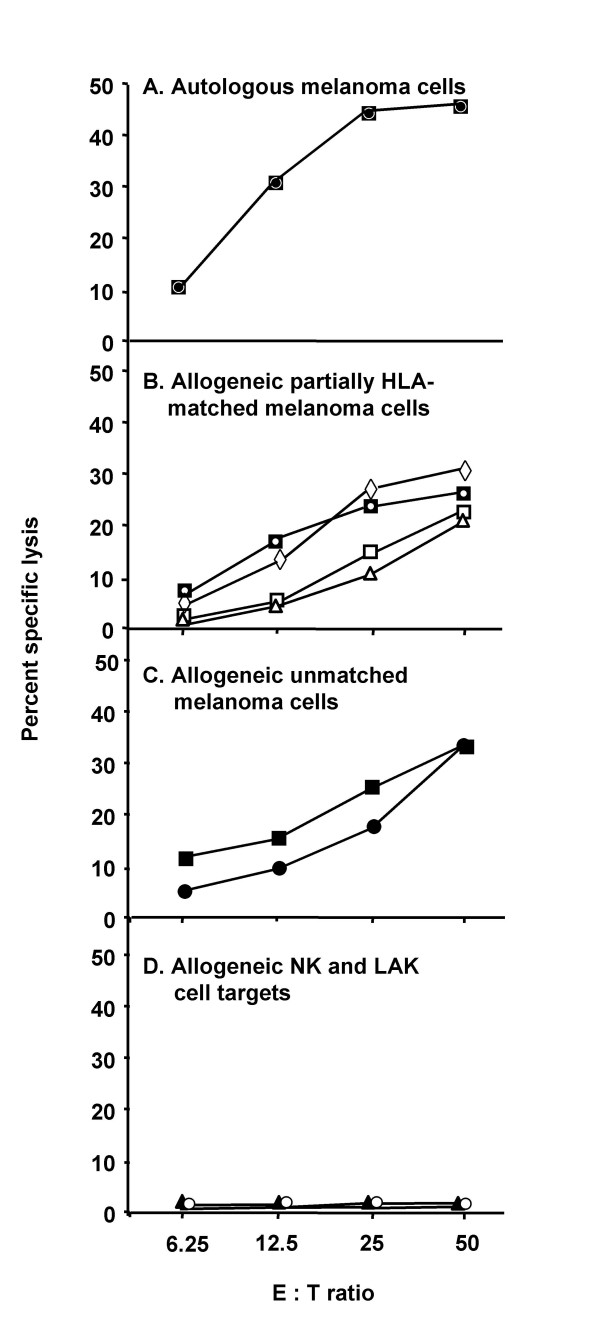
**Cytotoxic reactivity of CD8^+ ^CTL line against autologous melanoma cells**. CD8^+ ^CTL line was generated in MLTC by stimulating the PBMC of melanoma patient 793 with irradiated autologous WM793 melanoma cells. Cultures received IL-2 from day 3 onward, and were restimulated every 7 days for 2 months with autologous irradiated melanoma and EBV-B cells or PBMC. Cytotoxic effector cell responses were measured after 8 weeks. CTL lysis of ^51^Cr-labeled: A) autologous WM793 melanoma cells (○); B) allogeneic partially HLA-matched DM196 (□), ME9874 (Δ), A375 (▽), and WM75 (◇) melanoma cells; C) HLA-unmatched allogeneic melanomas WM164 (■) and WM1158 (●); D) NK cell target K562 (▲) lymphokine-activated killer cell target Daudi (◆) was determined in 4 h ^51^Cr-release assays using E:T ratios of 6.25–50.

**Figure 2:**
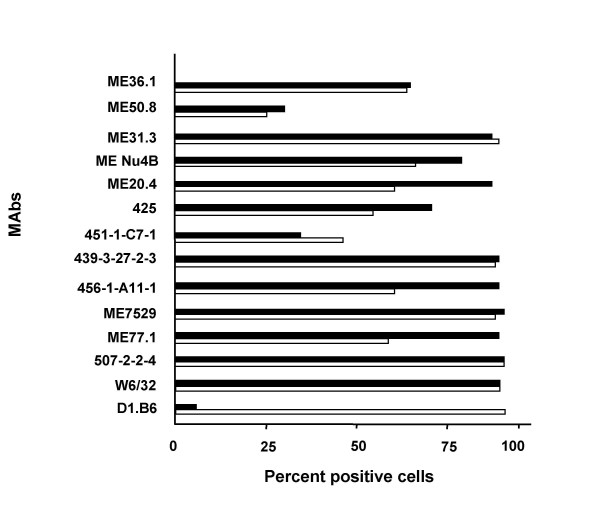
**Reactivity of anti-melanoma MAbs to melanoma cells. **Melanoma cells (5 × 10^4^) WM793 (□) and 1205LU (■) were incubated for 45 min with saturating concentrations of anti-melanoma MAbs, cells were washed to remove unbound MAbs and were further incubated with FITC conjugated anti-mouse antibody for 45 min. Cells were washed and binding of antibody was analyzed in the cytofluorograph. Results are expressed as percent positive cells.

The cytolytic activity of the CD8^+ ^CTL line against autologous and allogeneic partially HLA-matched and unmatched melanoma cells was tested at various E:T ratios (6.25–50) at 8 weeks in culture. As controls, NK susceptible target (K562) and LAK sensitive target (Daudi) were used. The CTL line lysed autologous WM793 melanoma cells as well as partially HLA-matched (DM196, ME9874, A375 and WM75) and HLA-unmatched allogeneic melanoma cells (WM164 and WM1158), but not NK-sensitive K562 or LAK-sensitive Daudi cells (Fig. 1). In contrast to autlogous WM793 melanoma cells, which were non-metastatic in nude mice and lysed by CTL 793, 1205LU metastatic variant cells of WM793 cells isolated from nude mice were not lysed by the CTL (Table II). Absence of 1205LU lysis by CTL793 cannot be explained by absence of the expression of known melanoma-associated antigens or HLA class I in 1205LU cells, compared to WM793 cells (Fig. 2). However, we cannot exclude the possibility that other antigens are differentially expressed by the two melanoma cell lines. Thus, the antigen recognized by the CTL may be expressed by primary (WM793), but not metastatic variant (1205LU) melanoma cells, derived from the same patient. However, expression of HLA class II was low (<5%) in 1205LU cells compared to WM793 cells (Fig. 2). Since CTL793 are CD8^+^, we do not expect HLA class II to play a role in WM793 lysis by the CTL. Furthermore, there is strong evidence that CTL793 are HLA unrestricted in their capacity to lyse autologous tumor cells (see below).

**Table 2:** Lysis of various HLA-matched and -unmatched target cells and IFN-γ secretion by CD8^+ ^793CTL

Stimulator and target cell		HLA subtype^a^	% Maximal	IFN-γ secretion	GM-CSF secretion
				
Designation	Origin		Cyto-toxicity^b^	(U/ml)^c^	(U/ml)
WM793	Autologous primary melanoma	**A1, A29, B57[17], B35, DRB1 11, DQB1 0301**	40.1^d^	10^d^	5.1^d^
1205LU^e^	Autologous metastatic variant	**A1, A29, B57[17], B35, DRB1 11, DQB1 0301**	6.6	<0.01	<0.01
DM196	Allogeneic metastatic melanoma	A23(9), Aw34, **B57[17]**, B44, DR1, DR3, DRw1, DRw52	12^d^	2.2^d^	2.5^d^
ME9874	Allogeneic metastatic melanoma	A2, A24, **B57[17]**, B60, Cw3, Cw7, DR7, DQ2	8.3^d^	5.9^d^	3.2^d^
A375	Allogeneic metastatic melanoma	**A1**, A2, **B57[17]**, C6	20.3^d^	41.2^d^	6.2^d^
WM75	Allogeneic metastatic melanoma	A2, **A29**, B12w44, DR4, DR7	24.2^d^	4.6^d^	2.0^d^
WM98	Allogeneic metastatic melanoma	**A1**, A3, B8, DR3	8.1	<0.01	<0.01
WM164	Allogeneic metastatic melanoma	A24, B7, C7, DR13, DQ1, DQ6, DRw52	29.8^d^	1.6	2.0^d^
WM1158	Allogeneic metastatic melanoma	A11, A24, B16, B60 (40), C3, DR13, DR4, DQ3, DQ6, DRw52, DRw53	45^d^	<0.01	<0.01
U87MG	Allogeneic glioma	A2, B7, B44, Cw05, Cw07, DRB1 03, DRB115, DQB1 02, DQB1 06	0	<0.01	N.D.
U373MG	Allogeneic glioma	A2, B18, Cw05, DRB103	0	<0.01	N.D.
MDAMB231	Allogeneic breast carcinoma	A2, B40, B41, Cw02, Cw17, DRB1 07, DRB1 13, DQB1 02, DQB1 03	0	<0.01	N.D.
WC007	Allogeneic colorectal carcinoma	**A1, **A3, B35, DR1, DR4	0	<0.01	N.D.
WC008	Allogeneic colorectal carcinoma	**A1, **A3, B15,**B57[17], **DR4	0	<0.01	N.D.
FOM708	Allogeneic melanocytes	**A1**, A11, B7, B8, C1, C6	3.7	<0.01	<0.01
FOM723	Allogeneic melanocytes	A3, **A29, **B7, B44	7.8	<0.01	<0.01
FOM1020	Allogeneic melanocytes	A2, A3, B37, **B57**, C6	5.2	<0.01	<0.01
793 fibroblasts	Autologous fibroblasts	**A1, A29, B57[17], B35, DRB1 11, DQB1 0301**	N.D.^f^	<0.01	N.D.
793 EBV-B	Autologous B cells	**A1, A29, B57[17], B35, DRB1 11, DQB1 0301**	0	1.3	N.D.
888EBV-B	Allogeneic B cells	**A1**, A24, B52, B55, C1, C7, DR15	N.D.	<0.01	N.D.
1363EBV-B	Allogeneic B cells	**A1**, A2, B44, B51, C1, DR1	N.D.	<0.01	N.D.
1088EBV-B	Allogeneic B cells	**A1**, A2, B8, B44, C5, DR4, DR17	N.D.	<0.01	N.D.
1102EBV-B	Allogeneic B cells	A2, A24, B55, B62, C3, DR4, DR15	N.D.	<0.01	N.D.
4226EBV-B	Allogeneic B cells	A24, A32, B27, B38, C3, DR4, DR15	N.D.	<0.01	N.D.
K562	NK-sensitive erythroleukemia cells	N.D.	0	<0.01	N.D.
Daudi	lymphokine-activated killer cell-sensitive lymphoma cells	N.D.	0	<0.01	N.D.

^a ^Based on tumor cell typing, except for WM75 and WM98 (based on lymphocyte typing). HLA types of WM793 cells and those matching between WM793 and other cells are in bold.

^b ^Lysis of labeled tumor target cells was determined in 4-hr ^51^Cr release assay using optimal E:T ratio of 20. Values represent means of triplicate determinations. Each cell line was tested in at least 2 different assays with similar results.

^c ^IFN-γ and GM-CSF secretions were determined by RIA. Values represent means of triplicate determinations. Each cell line was tested in at least 2 different assays with similar results.

^d ^p < 0.05 when compared to corresponding cpm values of spontaneous release of ^51^Cr by target cells and/or spontaneous release of cytokine by CTL.

^e ^Lung metastasis obtained in nude mice by injecting autologous WM793 cells s.c.[8]

^f ^N.D. = Not determined.

A larger panel of allogeneic HLA-matched and -unmatched melanoma, glioma, breast and colorectal carcinoma cells were used as targets in cytotoxicity assays. Of the eight allogeneic melanoma cells used as targets in CTL assays, six (DM196, ME9874, A375, WM75, WM164, and WM1158) were significantly (at p < 0.05 level) and consistently (in 2 different assays) lysed (Fig. 1, Table II). Four of the six allogeneic melanoma cell lines that were lysed by the CTL share HLA-A1, A29 and/or B57 [17] alleles with the autologous WM793 cells. Two of the cell lines (WM164 and WM1158) lysed by the CTL (Fig. 1, Table II) were HLA-unmatched. There was no significant lysis of allogeneic partially HLA-matched melanocytes (FOM708, FOM723, FOM1020), autologous 793 EBV-B cells, allogeneic HLA-unmatched glioma cells (U87MG and U373MG), breast carcinoma (MDAMB231) or colorectal carcinoma (WC007 and WC008) cells (Table II).

### Cytokine secretion of CD8^+ ^CTL793

Supernatant from CD8^+ ^CTL793 after 24–48 hr of stimulation with autologous melanoma cells WM793 contained significant amounts of IFN-γ, TNF-α, and GM-CSF, but not IL-2 or IL-4 (data not shown, except for IFN-γ release in Table II). IFN-γ, TNF-α, and GM-CSF were produced by the CTL, and not by irradiated autologous melanoma cells, since the supernatants derived from the melanoma cells showed no significant cytokine secretion (data not shown). To determine the specificity of cytokine release by the CTL, IFN-γ and GM-CSF production by the CTL after stimulation with various tumor and non-tumor cells was determined (Table II). All six allogeneic, partially HLA-matched melanoma cells, (DM196, ME9874, A375, WM75, WM164 and WM1158) induced significant IFN-γ and GM-CSF production by the CTL and also were lysed by the CTL (Table II). None of the cell lines derived from gliomas, breast and colorectal carcinomas, or melanocytes, fibroblasts or EBV-B lymphocytes induced IFN-γ release by the CTL (Table II).

### Blocking of CTL activity of CD8^+ ^CTL793 using various MAb

Melanoma cells WM793 express HLA-class I and II (97% and 74% of the cells positive, respectively; Table I). CTL activity against autologous WM793 cells was not blocked by pre-incubating target cells with saturating concentrations of MAb to HLA-class I (W6/32) or class II (MAb B33.1 or MAb D1.B6) (Table III), indicating that CTL activity is HLA-unrestricted. Surprisingly, these MAbs enhanced tumor cell lysis (Table III). In our related studies, these MAbs were able to block the tumor target cell lysis of other HLA-restricted CTLs [10,13]. CTL activity against partially matched allogeneic DM196 melanoma cells was not blocked by MAb to HLA class I (data not shown), indicating that CTL activity is HLA unrestricted.

**Table 3:** Absence of Cytotoxicity blocking of CD8^+ ^793 CTL line against WM793 by MAbs

MAb^a^			% WM793 target cell lysis	% Inhibition of WM793 target cell lysis^b^
		
Designation	Specificity	Isotype		
W6/32	HLA class I	IgG2a	27.9	-47.6
B33.1	HLA class II	IgG2a	23.8	-26.0
D1.B6	HLA class II	IgG1	21.0	-11.3
DX2	FAS (CD95)	IgG1	19.9	-5.3
OKT3	CD3	IgG2a	5.2	72.5^c^
HI149	CD1	IgG1	23.6	-28.9
H24B5^d^	Influenza virus	IgG2a	18.9	2.6
Normal mouse Ig^d^	Unknown	Predominantly IgG1	18.3	3.2

^a ^Tumor targets were incubated with MAb (saturating concentrations, 10 μg/ml) for 1 hr at room temperature and excess MAb was removed before the 4-hr ^51^Cr-release assay.

^b ^Negative values indicate enhancement of lysis. Means of triplicate determinations at E:T ratios of 2.

^c ^Value significantly (p < 0.01) different when compared to isotype matched control antibody.

^d ^Isotype-matched control antibodies.

CTL793 express FAS-L and WM793 melanoma cells express FAS (Table I). To determine the role of FAS and FAS-L interaction in 793 CTL-mediated lysis of WM793 tumor targets, tumor cells were incubated with anti-FAS MAb DX2 before the CTL activity against the cells was tested. Anti-FAS MAb was unable to block 793 CTL lysis of WM793 target cells (Table III), indicating that CTL killing is independent of the FAS and FAS-L mediated pathway.

Low percentage of WM793 cells expresses CD1 (Table I). The role of CD1 in CTL793-mediated lysis of WM793 tumor targets was tested by incubating tumor cells with anti-CD1 MAb HI149 before the CTL activity against the cell was tested. Anti-CD1 MAb was unable to block 793 CTL lysis of WM793 target cells, indicating that CD1 is not involved in CTL-mediated lysis of tumor cells (Table III).

CTL activity of 793 CTL against WM793 cells was significantly blocked by preincubating effector cells with saturating concentrations of anti-CD3 MAb (Table III). Thus, lysis of target cells is mediated by TCR.

## Discussion

There are a number of reports of HLA-unrestricted CD8^+ ^CTL derived from the lymphocytes of melanoma patients [3-6,14]. Kubo *et al *[14] described two CTL clones, each expressing a single TCR (αβ) and recognizing HLA-matched tumor targets in an HLA-restricted fashion and HLA-unmatched targets in an HLA-unrestricted manner. HLA-unrestricted CTL from ovarian, breast and pancreatic carcinoma patients also have been reported and they are known to recognize epitopes of mucin (MUC1) tumor-associated antigen [15-17]. However, it is unclear whether the CTL against melanomas and carcinomas are truly HLA-unrestricted, as target cells were not fully HLA subtyped [3-6] and high E:T cell ratios were used in cytolysis assays [5,6] (see Introduction).

Our study provides strong evidence that the cytolytic activity of the CD8^+ ^793 CTL line is HLA-unrestricted. We have tested our CTL in two sets of experiments to confirm that the cytolytic activity is HLA-unrestricted. In the first set of experiments we have used a panel of HLA-matched and -unmatched melanoma targets in CTL lysis assays (Table II). The target cells were fully HLA subtyped. In the second set of experiments we have used anti-HLA antibodies to block the cytolytic activity of the CTL at low E:T cell ratios (Table III). CD8^+ ^793 CTL line lysed autologous melanoma cells, and four different partially HLA-matched (HLA-A1, A29 or HLA-B57 [17] alleles) and two unmatched allogeneic melanoma cells; none of the allogeneic tumor cell lines derived from glioma, breast or colon carcinoma were lysed. These results suggest that the CD8^+ ^CTL lysed melanoma targets in an HLA-unrestricted manner. These results were confirmed in cytokine release assays and in assays where autologous tumor cells were incubated with MAb to HLA-class I or -class II prior to determining tumor cell lysis by the CTL at low E:T ratios. Unexpectedly, pre-incubation of tumor cells with MAb to HLA-class I or -class II resulted in enhancement of CTL tumor lysis (Table III). Similar enhancement of CTL target cell lysis in the presence of anti-HLA antibodies was observed by other investigators [3,4], although the mechanism of this phenomenon is unknown.

Our data rule out a role of Fas/Fas ligand interaction in tumor cell lysis by the CD8^+ ^CTL line as MAb to FAS was unable to block CTL lysis of melanoma cells in spite of expression of FAS-L and FAS by the CTL and tumor cells, respectively (Table I).

The CD8^+ ^CTL described here recognize an antigen(s) preferentially expressed by melanoma cells. This antigen was not present on allogeneic cell lines of glioma (two), breast carcinoma (one), and colorectal carcinoma (two), or allogeneic partially matched melanocytes (three) or autologous fibroblasts (one), and autologous (one) or allogeneic (five) EBV-B cells. A presumably HLA-unrestricted CTL clone recognizing a determinant shared by allogeneic melanoma and lung carcinoma cells has been described [14]. A short-term presumably HLA-unrestricted CTL line established by repeated stimulation of lymph node T cells obtained from a pancreatic carcinoma patient recognized and lysed MUC-1 positive pancreatic, breast, and ovarian carcinoma cells[15,17]. To our knowledge, MUC-1 is not expressed by melanoma cells, and thus it is highly unlikely that MUC-1 is the candidate antigen responsible for CD8^+ ^CTL-mediated lysis.

Identification of the melanoma-associated antigen recognized by the CD8^+^, HLA-unrestricted CTL described here will provide a vaccine for a broader population of melanoma patients. The antigen is expressed by the majority of allogeneic, metastatic melanoma cells, although it is lost by metastatic variant cells isolated from primary, autologous WM793 melanoma cells by passaging these cells in nude mice.

## List of abbreviations

CTL, cytotoxic T cell; EBV, Epstein-Barr virus; E:T, effector-to-target; FITC, fluorescein isothiocyanate; GM-CSF, granulocyte monocyte-colony stimulating factor; HLA, human leukocyte antigen; IFN-γ, interferon gamma; IL, interleukin; LAK, lymphokine-actived killer; MAb, monoclonal antibody; MLTC, mixed lymphocyte tumor culture; MUC1, mammary/pancreas mucin; PBMC, peripheral blood mononuclear cells; PE, phycoerythrin; RIA, radioimmunoassay; RT-PCR, reverse transcriptase-polymerase chain reaction; TCR, T-cell receptor; TNF, tumor necrosis factor.
